# *Parathyridaria percutanea* and Subcutaneous Phaeohyphomycosis

**DOI:** 10.3201/eid2509.190383

**Published:** 2019-09

**Authors:** Shivaprakash M. Rudramurthy, Megha Sharma, Nandini Sethuraman, Pinaki Dutta, Bansidhar Tarai, Jayanthi Savio, Amanjit Bal, Usha Kalawat, Arunaloke Chakrabarti

**Affiliations:** Postgraduate Institute of Medical Education and Research, Chandigarh, India (S.M. Rudramurthy, M. Sharma, N. Sethuraman, P. Dutta, A. Bal, A. Chakrabarti);; Apollo Hospitals, Chennai, India (N. Sethuraman); Max Super Speciality Hospitals, New Delhi, India (B. Tarai);; St. Johns Medical College and Research Institute, Bengaluru, India (J. Savio);; Sri Venkateshwara Institute of Medical Sciences, Tirupati, India (U. Kalawat)

**Keywords:** nonsporulating fungi, dematiaceous fungi, Pleosporales, Parathyridaria percutanea, phaeohyphomycosis, fungi, India

## Abstract

*Parathyridaria percutanea* is an emerging fungus causing subcutaneous phaeohyphomycoses in renal transplant recipients in India. We identified *P. percutanea* from a patient with subcutaneous phaeohyphomycosis. From our culture collection, we identified the same fungus from 4 similar patients. We found 5 cases previously described in literature.

*Parathyridaria percutanea,* earlier known as *Roussoella percutanea* in the order *Pleosporales,* has been reported to cause subcutaneous phaeohyphomycoses (*1*,*2*). *P. percutanea* belongs to coelomycetes, a group of fungi in which the conidia or asexual propagules lie within a cavity. *Parathyridaria* spp. generally exist as plant saprobes; *P. percutanea* is the only species reported as an opportunistic pathogen. 

We recently observed a case of subcutaneous phaeohyphomycosis caused by *P. percutanea.* The patient was a 33-year-old man who had ACTH-dependent Cushing’s disease with 2 cutaneous lesions, one under the left axilla and the other on the ulnar aspect of the left forearm, that had progressed slowly over 3 years (Appendix Figure 1, panel A). Direct microscopy of a biopsy sample taken from the left forearm lesion revealed dematiaceous septate hyphae with irregular hyphal swellings (Appendix Figure 1, panel B). Colonies on Sabouraud’s dextrose agar at 25°C were flat, spreading with sparse aerial hyphae after 1 week, and later turned to cottony greenish-black growth (Appendix Figure 1, panel C). Lactophenol cotton blue mount revealed nonsporulating dematiaceous hyphae with chlamydospores (Appendix Figure 1, panel D). Several attempts to induce sporulation (on oatmeal agar and malt extract agar) failed. Histopathologic examination (Appendix Figure 1, panels E–G) showed neutrophilic infiltration with fungal hyphae, nodular swellings on Giemsa stain, and black hyphae on Grocott-Gomori’s methamine silver stain.

We identified the fungus as *Roussoella percutanea* of the order *Pleosporales,* later renamed *P. percutanea,* by PCR sequencing of the internal transcribed spacer (ITS) and 28S regions of ribosomal DNA, as described previously (*3*)*.* ITS sequencing of our strain NCCPF104001 (GenBank accession nos. MG708109 [by ITS] and MG708116 [by 28S]) had 99.8% identity with CBS128203 (type strain, GenBank accession no. KF322117) and CBS868.95 (GenBank accession no. KF322118), whereas 28S sequences had 100% identity with CBS128203 (GenBank accession no. KF366448) and CBS868.95 (GenBank accession no. KF366449) (Appendix Figure 2, panels A. B). The patient refused further treatment in the hospital and left against medical advice.

We screened all the isolates deposited in our National Culture Collection of Pathogenic Fungi (NCCPF, Chandigarh) and characterized them phenotypically as *Pleosporales*. Of 7 such isolates, we identified 4 as *P. percutanea* by sequencing (Table). We further subjected these isolates to phylogenetic analysis of ITS and large ribosomal subunit (28S) of the rDNA using MEGA software version 6 (https://megasoftware.net) (*3*). The strains identified as *P. percutanea* clustered together with the ITS and 28S sequences of CBS12608 and CBS868.95 strains, the other 2 *P. percutanea* isolates reported with gene sequences (*2*). Phylogenetically, *Parathyridaria* is now a distinct genus and clearly separated from closely related genera such as *Roussoella* and *Thyridaria* (Appendix Figure 2, panels A, B). 

**Table:** Comparison of demographic, clinical, and microbiological features of subcutaneous phaeohyphomycosis due to *Parathyridaria percutanea*

Case no.	Study or isolate ID	Age, y/ sex	Residing country; native country	Risk factor (time since)	Site	Diagnostic modality	Molecular targets	GenBank accession nos., ITS/28S	AFST	Treatment	Outcome
Previously reported											
1	(*5*)†	63/M	The Netherlands; Aruba, Caribbean	Renal tx (2 y)	Rt lateral foot	Culture	Misidentified as *M. mycetomatis* ITS	KF322118/ KF366449	Low MIC to VR, PS, IT†	Multiple aspiration of pus + surgical excision	Healed, no recurrence
2	(*2*)	45/M	United States; India	None; tattoo on the site‡ (2 y); DM	Lt lateral foot and ankle	Culture	ITS	KF322117/ KF366448	Low MIC to VR, PS, IT	Azole therapy for 4 mo + surgical excision§	LTFU
3	(*6*)	65/M	France; Congo	Renal tx (1 y)	Lt lateral malleoli	HPE; No culture sent	NA¶	NA	ND	PS + surgical excision	ND
4	(*7*)	55/F	Germany; Somalia	Renal and pancreatic tx (3 y)	Lt knee synovial bursitis	GMS staining and culture	ITS	NA	Low MIC to azole VR, PS, IT	VR for 1 mo + surgical excision	Healed, No recurrence
5	(*8*)	47/M	United Kingdom; India	Renal tx (1.5 y)	Rt Achilles	Calcofluor and PAS stain	ITS, 28S	NA	ND	VR for 2 weeks + surgical excision	Healed. No recurrence
Reported in this study											
6	NCCPF 104001 (index case)	35/M	India (Chandigarh); India	ACTH-dependent Cushing’s syndrome	Lt forearm and axilla	KOH, Calcofluor and culture	ITS, 28S	MG708109/ MG708116	NCO	None	LTFU
7	NCCPF 104003; (*4*)#	47/M	India (Andhra Pradesh); India	Post–renal transplant	Great toe	KOH and culture	ITS, 28S	MG708107/ MG708115	NCO	VR	Healed, no recurrence
8	NCCPF 104004	54/F	India (Delhi); India	Interstitial lung disease on steroids	Elbow, knee	KOH and culture	ITS, 28S	MG708106/ MG708114	NCO	Surgical excision and VR	Healed, no recurrence
9	NCCPF 104005	45/M	India (Andhra Pradesh); India	Post–renal transplant	Foot	KOH and culture	ITS, 28S	MG708105/ MG708113	NCO	None	LTFU
10	NCCPF 104006	50/M	India (Bangalore); India	Post–renal transplant	Rt foot	KOH and culture	ITS, 28S	MG708108/NA	NCO	None	LTFU

*AFST, antifungal susceptibility testing; DM, diabetes mellitus; GMS, Grocott methenamine silver; HPE, histopathological examination; IT, itraconazole; ITS, internal transcribed spacer; Lt, left; LTFU, lost to follow-up; MIC, minimal inhibitory concentration; NA, not applicable; NCCPF, National Culture Collection for Pathogenic Fungi; NCO, not carried out; ND, not documented; PAS, periodic acid–Schiff; PS, posaconazole; Rt, right; Tx, transplant; VR, voriconazole. †Ahmed et al. (*2*) studied the culture originally reported by Meis et al (*5*). ‡Immunocompetent patient with a tattoo at the site of infection; diabetes mellitus was diagnosed incidentally when patient sought care. §Posaconazole was discontinued (due to gastrointestinal intolerance) and switched to voriconazole, which was also discontinued (due to visual disturbances) and changed to itraconazole. ¶Panfungal PCR done but no targets mentioned (*6*).  #Case described earlier as an “uncommon black fungus belonging to order *Pleosporales*” (*10*) was identified at NCCPF. This case is described again in the study series as *P. percutanea*.

We searched published literature on Medline and PubMed for subcutaneous phaeohyphomycoses caused by *P. percutanea* or *R. percutanea* and identified 5 cases (Table). All 5 patients originated from tropical countries including the Caribbean islands (*5*), Republic of the Congo (*6*), Somalia (*7*), and India (*2*,*8*). Including these 5 with the case-patients we identified from culture and our study patient, 7 of 10 total cases originated in India. The patients had lesions in the extremities; we expect that the fungus is present in our environment and gains access from traumatic inoculation of patients working in the field or walking barefoot. The clinical description of all 10 patients is presented in the Table. Male patients outnumbered female patients. In 2 patients, underlying muscle tendon (*2*) and joint bursa (*7*) were involved. No discharging sinus or granuloma formation was seen in any of the 10 patients. 

*P. percutanea* infection occurred in immunosuppressed patients; 9/10 patients were either renal transplant recipients (7 patients) or on steroid therapy (2 patients). The disease manifested 1–3 years posttransplant. Incidence of subcutaneous phaeohyphomycoses is reported in <3.6% of renal transplant recipients (*10*). The tenth patient had diabetes, and the infection of *P. percutanea* occurred at a tattoo site, manifesting 2 years after tattooing. The fungus may remain dormant in subcutaneous tissue after traumatic inoculation and multiply slowly at the opportune time when host immunity is depressed because of immunosuppression or diabetes.

The outcome of *P. percutanea* infection was known in 5/10 patients, and they responded to surgical resection of the lesion followed by voriconazole therapy. The joint guidelines of the European Society of Clinical Microbiology and Infectious Diseases Fungal Infection Study Group and the European Confederation of Medical Mycology on the management of subcutaneous phaeohyphomycosis (*9*) recommended surgical resection (recommendation AII) along with oral azoles in immunosuppressed patients to prevent dissemination of disease (recommendation BIII). In vitro susceptibility testing, conducted for 3 isolates by Ahmed et al. (*2*) and Almagro-Molto et al. (*7*), revealed that *P. percutanea* exhibited low MIC to itraconazole, voriconazole, and posaconazole (Table). Therefore, itraconazole and posaconazole can be used in patients receiving other liver-metabolized drug therapies.

Especially in renal transplant patients in India, *P. percutanea* could be a possible etiologic agent of subcutaneous phaeohyphomycosis. Sequencing of ITS and 28S regions of ribosomal DNA confirms diagnosis. Effective treatment could include surgical excision of lesions and voriconazole or posaconazole therapy.

Appendix Additional information about *Parathyridaria* infection causing subcutaneous phaeohyphomycosis.
