# Supplementary material for: Parathyridaria percutanea and Subcutaneous Phaeohyphomycosis
Source: Emerg Infect Dis. 2019 Sep;25(9):1768–9. doi: 10.3201/eid2509.190383 (PMC6711204; doi:10.3201/eid2509.190383)
Supplement: Appendix — Additional information about Parathyridaria infection causing subcutaneous phaeohyphomycosis. [file 19-0383-Techapp-s1.pdf]

Article DOI: https://doi.org/10.3201/eid2509.190383

Parathyridaria percutanea and Subcutaneous Phaeohyphomycosis

Appendix

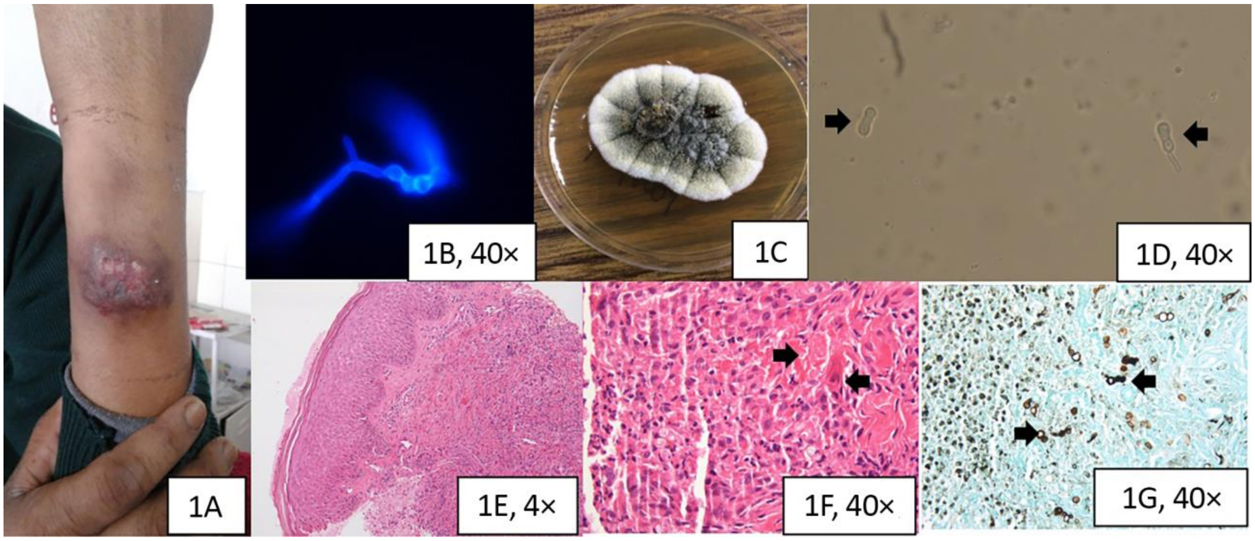

Appendix Figure 1. A) Hyperpigmented swelling with irregular ulcerated surface on dorsal aspect of left forearm. B) Calcoflour stain showing fluorescing septate hyphae with nodular swellings; original magnification ×40. C) Greenish-black colony after 2 weeks of incubation. D) Lactophenol cotton blue mount showing nonsporulating hyphae with chlamydospores; original magnification ×40. E) Histopathological examination of the biopsy showing neutrophilic infiltration in the subcutaneous tissue; original magnification ×4. F) The same area showing presence of fungal hyphae' original magnification ×40. G) Grocott-Gomori's methamine silver stain showing melanized fungal elements; original magnification ×40.

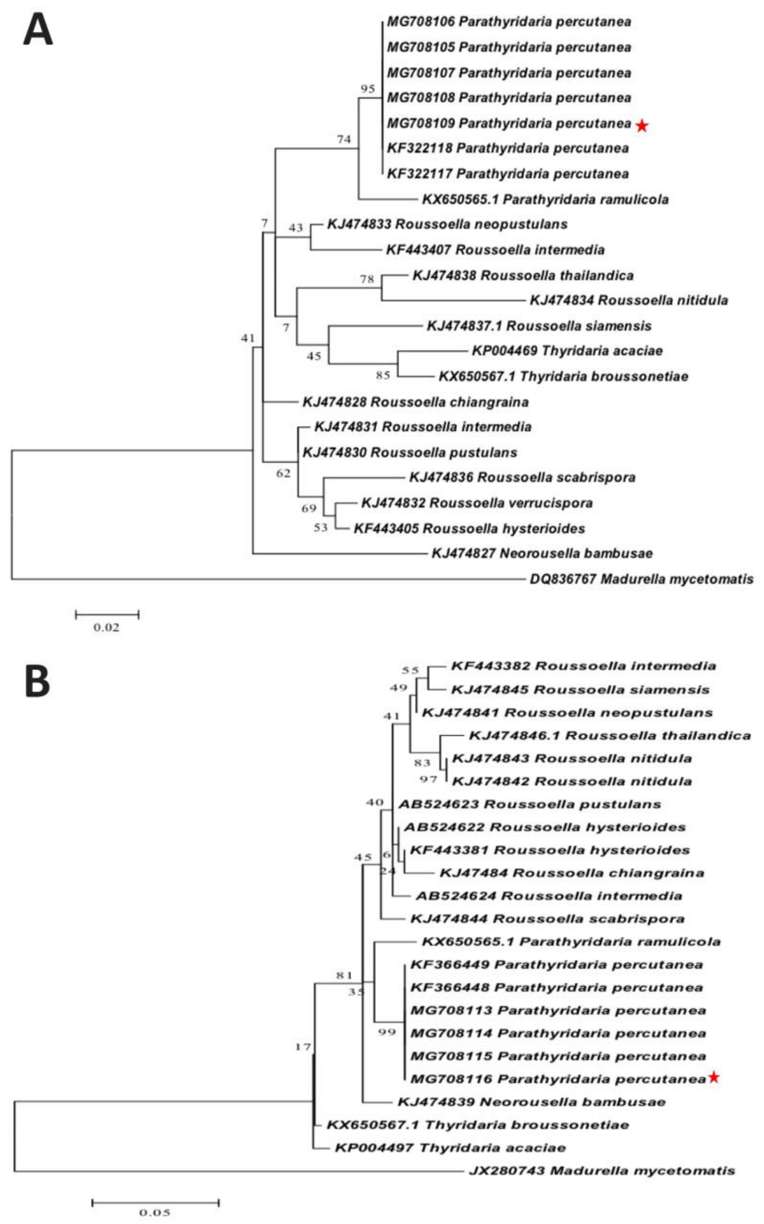

Appendix Figure 2. Molecular phylogenetic analysis of ITS (A) and 28S (B) sequences of rDNA by maximum-likelihood method. The evolutionary history was inferred by maximum likelihood method based on the Tamura-Nei model. The percentage of trees in which the associated taxa clustered together is shown next to the branches. Initial tree(s) for the heuristic search were obtained automatically by applying neighbor-join and BioNJ algorithms to a matrix of pairwise distances estimated using the maximum composite likelihood approach, and then selecting the topology with superior log likelihood value. The tree is drawn to scale, with branch lengths showing number of substitutions per site. All positions containing gaps and missing data were eliminated. Red star indicates the sequence representing the index case, which clustered together with other *P. percutanea* isolates from our culture collection (named MG) and 2 isolates already in the database (named KF). ITS, internal transcribed spacer.
